# Associations of positive and negative social connections with brain health in UK Biobank data

**DOI:** 10.1002/alz.091057

**Published:** 2025-01-09

**Authors:** Suraj Samtani, Gowsaly Mahalingam, Wei Wen, Henry Brodaty, Perminder S. Sachdev

**Affiliations:** ^1^ Centre for Healthy Brain Ageing (CHeBA), University of New South Wales, UNSW Sydney, NSW Australia; ^2^ Centre for Healthy Brain Ageing (CHeBA), UNSW Sydney, Sydney, NSW Australia

## Abstract

**Background:**

Social connections are linked to brain health. Studies to date have ignored negative social connections and the association between social connections and white matter health. We explored the relationship between positive *and* negative social connections and brain imaging including white matter health. We hypothesized that positive and negative social connections would be related to better and worse brain health, respectively.

**Method:**

We analysed UK Biobank data from 5704 adults aged 40+ (M_age_ = 54.12, 51.19%) who had brain scans at waves 2 and 3. We examined baseline social connections which were positive (household size, friend/family visits, community group engagement, having a confidante) and negative (loneliness, belittling or violence in romantic relationships). Outcome variables were total grey and white matter, amygdala and hippocampus volumes, white matter integrity and hyperintensities, and peak width of skeletonized mean diffusivity (PSMD; a marker of microstructural white matter changes) at waves 2 and 3. We ran linear mixed models controlling for age, sex and scanner (partially adjusted) and additionally for intracranial volumes (for brain volumes), education, physical activity, BMI, depression, hearing loss, alcohol use, smoking, hypertension, CVD, and air pollution (fully adjusted).

**Result:**

In fully adjusted models, having a larger social network was associated with less decline from wave 2 to 3 in total grey matter (B = 671.61, 95%CI: 226.96,1,115.85), white matter (B = 511.72, 95%CI: 52.33,971.82), and right hippocampus volumes (B = 8.99, 95%CI: 1.44,16.54). Living with more people (B = 0.00049, 95%CI: 0.00021, 0.00077) and having a larger network size (B = 0.00035, 95%CI: 0.00024, 0.00045) were associated with less decline in white matter integrity. Experiencing romantic partner violence was associated with a greater increase in PSMD (B = 0.000079, 95%CI: 0.000016, 0.00014). Loneliness was not associated with brain health changes.

**Conclusion:**

In a longitudinal sample of adults aged 40+, having a larger social network was associated with better brain health, including less decline in hippocampal volumes and white matter integrity. Experiencing romantic partner violence was associated with microstructural white matter changes. Our results highlight the need to screen for positive and negative social connections in adults in mid and late life to help maintain good brain health.

